# The FMRP regulon: from targets to disease convergence

**DOI:** 10.3389/fnins.2013.00191

**Published:** 2013-10-24

**Authors:** Esperanza Fernández, Nicholas Rajan, Claudia Bagni

**Affiliations:** ^1^Center for the Biology of Disease, Vlaams Institut voor BiotechnologieLeuven, Belgium; ^2^Center for Human Genetics, Leuven Institute for Neuroscience and Disease, KU LeuvenLeuven, Belgium; ^3^Department of Biomedicine and Prevention, University “Tor Vergata”Rome, Italy

**Keywords:** fragile X syndrome, autism, schizophrenia, major depressive disorders, FMRP, RNA-binding proteins, synaptic plasticity, local protein synthesis

## Abstract

The fragile X mental retardation protein (FMRP) is an RNA-binding protein that regulates mRNA metabolism. FMRP has been largely studied in the brain, where the absence of this protein leads to fragile X syndrome, the most frequent form of inherited intellectual disability. Since the identification of the FMRP gene in 1991, many studies have primarily focused on understanding the function/s of this protein. Hundreds of potential FMRP mRNA targets and several interacting proteins have been identified. Here, we report the identification of FMRP mRNA targets in the mammalian brain that support the key role of this protein during brain development and in regulating synaptic plasticity. We compared the genes from databases and genome-wide association studies with the brain FMRP transcriptome, and identified several FMRP mRNA targets associated with autism spectrum disorders, mood disorders and schizophrenia, showing a potential common pathway/s for these apparently different disorders.

## Introduction

Protein synthesis at subcellular sites is a well-conserved mechanism that allows the rapid expression of specific genes in response to localized cues (Xing and Bassell, 2013). During transport, mRNAs are stabilized via association with multiple and different *trans*-acting factors, such as RNA-binding proteins (RBPs) and non-coding RNAs, forming ribonucleoparticles (RNPs) that vary in size and composition during cell cycle and development.

In highly polarized cells, such as neurons, mRNAs are transported from the nucleus to dendrites and axons where these molecules undergo local translation and degradation (Steward and Schuman, 2003; Bramham, 2008; Cajigas et al., 2010; Doyle and Kiebler, 2012; Hornberg and Holt, 2013) according to their subcellular localization and cellular inputs (Bramham, 2008).

RNA-binding proteins recognize and bind mRNA targets through regulatory elements in the 5′ and 3′ untranslated regions (UTRs) (Pichon et al., 2012), and in some cases the coding regions are also involved in these interactions (Anko and Neugebauer, 2012). Binding to mRNAs is mediated through well-known RNA-binding motifs, which are often present in multiple copies (Clery et al., 2008) and typically bind short RNA sequences (Anko and Neugebauer, 2012). Several RBPs cooperate for the binding of mRNA, thereby increasing the specificity of this interaction (Matlin et al., 2005; Ule and Darnell, 2006). The actin cytoskeleton might well facilitate RNA recognition, as this structure associates with RBPs and coordinates the binding of these proteins to mRNA (Percipalle, 2009). However, individual RBPs bind to several mRNAs. The multi-targeted binding property of RBPs has led to a model of regulated gene expression in eukaryotes termed “*the post-transcriptional operon*” (Keene, 2007).

The fragile X mental retardation protein (FMRP) is a widely studied RBP in the brain. Silencing of the *FMR1* gene encoding FMRP leads to fragile X mental retardation syndrome (FXS), the most common cause of inherited intellectual disability (Bagni et al., 2012). A majority of the clinical cases of FXS reflect a lack of FMRP due to a large trinucleotide CGG-repeat expansion in the 5′ UTR of the gene, resulting in *FMR1* gene silencing. Rare cases have been reported to carry partially deleted or mutated FMRP (De Boulle et al., 1993; Mila et al., 2000; Coffee et al., 2008; Collins et al., 2010). The *FMR1* gene and FMRP have also been associated with the pathogenesis of other disorders, such as fragile X-associated tremor ataxia syndrome (FXTAS), premature ovarian failure (POF), and autism spectrum disorder (ASD) (Bagni et al., 2012).

Here, we briefly reviewed the structure and function of FMRP, a multifunctional RBP that regulates the transport, stability and local protein synthesis of hundreds of RNAs in the brain. We further discuss how anomalies in the expression of FMRP alter the condition of its targets and ultimately, highlight a subset of FMRP target mRNAs dysregulated in autism spectrum disorders (ASDs), mood disorders (MDs) including bipolar disorder (BD), major depressive disorder (MDD), attention deficit hyperactive disorder (ADHD), and schizophrenia (SCZ).

## Fmrp structure, RNA targets and protein partners

The human *FMR1* gene is ubiquitously expressed (https://www.genevestigator.com/gv/), with higher abundance in some tissues (Kaufmann et al., 2002; Xie et al., 2009). The gene comprises 17 exons spanning 38 kb of Xq27.3 (Eichler et al., 1993). Alternative splicing of the gene results in the generation of 12 protein isoforms (De Boulle et al., 1993; Brackett et al., 2013).

In the mammalian brain, FMRP targets hundreds of mRNAs (Miyashiro et al., 2003; Darnell et al., 2011; Bagni et al., 2012; Gross et al., 2012; Wang et al., 2012) and non-coding RNAs, such as the brain cytoplasmic RNA BC1/BC200 *in vitro* and *in vivo* (Zalfa et al., 2003, 2005; Gabus et al., 2004; Johnson et al., 2006; Lacoux et al., 2012) and a few microRNAs (Jin et al., 2004; Edbauer et al., 2010; Gessert et al., 2010; Muddashetty et al., 2011; Tian et al., 2013).

**Figure 1:**
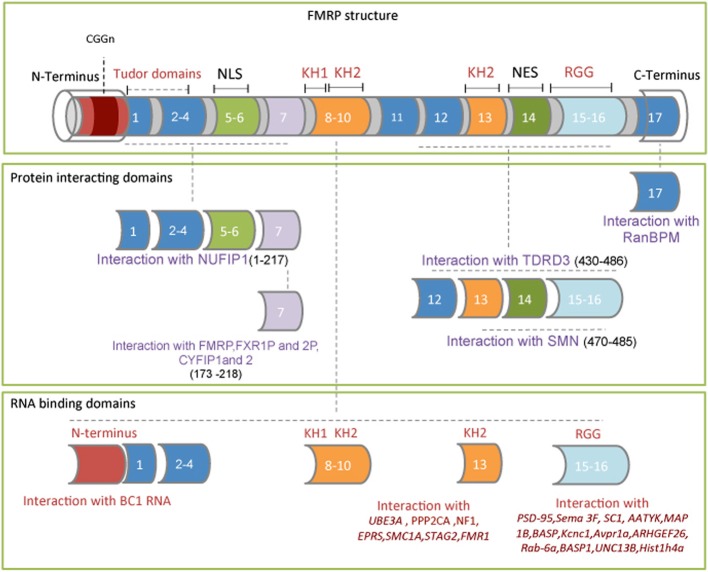
**FMRP exon structure comprising its functional domains. Upper frame:** The red box at the N-terminus of exon 1 indicates the location of the CGG triplet repeat within the 5′ UTR of the mRNA. The four RNA binding domains are: the N-terminus, the two K homology domains (KH1 and KH2) and the RGG box. **Middle frame:** FMRP domains interacting with NUFIP1, CYFIP1, CYFIP2, FXR1P, FXR2P, TDRD3, and SMN proteins. The FMRP amino acid sequence involved in these interactions is shown between the brackets. The nuclear localization signal (NLS) and the nuclear export signal (NES) are also indicated. **Lower frame:** The FMRP RNA binding domains and the RNA/mRNA targets directly bound are indicated.

Structural studies of the FMRP domain have contributed to the understanding of the molecular function/s of this protein. The N-terminal region, characterized by the presence of two Tudor domains (TD)(Ramos et al., 2006), binds *in vitro* RNA homopolymers and the small non-coding *BC1* RNA (Gabus et al., 2004; Zalfa et al., 2005; Lacoux et al., 2012) (Figure 1). The central region contains two K homology domains (KH) and a nuclear export signal (NES) (Valverde et al., 2008). The most severe single point mutation identified in a patient with FXS is an lle367Asn, located on helix α2 of the KH2 domain (De Boulle et al., 1993). The murine FMRP, carrying the corresponding mutation (Ile304Asn), loses the ability to bind RNA (Zang et al., 2009), likely reflecting the destabilization of the hydrophobic core, which partially unfolds the domain (Di Marino et al., 2013). A recent study in non-neuronal cells has shown that the FMRP Ile304Asn mutation reduces the binding affinity of a subset of mRNAs, such as *neurofibromatosis type 1* (*NF1*), *FMR1*, *bifunctional glutamate/proline-tRNA ligase* (*EPRS*), *serine/threonine-protein phosphatase 2A catalytic subunit alpha isoform* (*PPP2CA*), *ubiquitin-protein ligase E3A (UBE3A), structural maintenance of chromosomes protein 1A* (*SMC1A*) and *cohesin subunit SA-2* (*STAG2*) (Ascano et al., 2012).

The C-terminal region, containing an RGG box, is involved in the interaction of well-characterized FMRP mRNA targets (Darnell et al., 2001; Menon and Mihailescu, 2007; Westermark and Malter, 2007; Zalfa et al., 2007; Menon et al., 2008; Blackwell et al., 2010). The C-terminal region of FMRP binds *post synaptic protein-95* (*PSD-95*) *mRNA* (Zalfa et al., 2007), *microtubule associated protein 1B* (*MAP1B*) (Darnell et al., 2001; Zalfa et al., 2003), *semaphorin 3F* (*SEMA3F*) (Menon and Mihailescu, 2007), *extracellular matrix protein 2* (*SC1*), *brain acid soluble protein 1 (NAP22) (Darnell et al., 2001)* and *serine/threonine-protein kinase LMTK1 (AATYK)* (Blackwell et al., 2010) mRNAs, a few of which are depicted in Figure 1.

FMRP homodimerises and interacts with several cytoplasmic and nuclear proteins involved in mRNA metabolism and cytoskeleton-remodeling proteins (Bagni and Klann, 2012). Among the best characterized FMRP-interacting proteins are protein argonaute-2 (Ago2) (Muddashetty et al., 2011), 58 kDa microspherule protein (MSP58) (Davidovic et al., 2006), nuclear fragile X mental retardation-interacting proteins 1 and 2 (NUFIP1 and 2) (Bardoni et al., 2003), the survival of motor neuron (SMN) (Piazzon et al., 2008), the Tudor domain-containing protein 3 (Linder et al., 2008), nuclear export factor 2 (NXF2) (Zhang et al., 2007), dicer (Cheever and Ceman, 2009), cytoplasmic interacting protein CYFIP1 (Schenck et al., 2001, 2003; Napoli et al., 2008; De Rubeis et al., 2013) and the two paralogs, fragile X-related proteins 1 and 2 (FXRP1 and FXRP2) (Tamanini et al., 1999) (Figure 1).

## Cellular and molecular functions of FMRP

Although in neurons, FMRP has been localized in the nucleus, cell body and dendrites (Willemsen et al., 1996), the cytoplasmic function of FMRP has been the most studied. FMRP forms large cytoplasmic RNPs containing several proteins and RNAs, and this protein is involved in the transport, stability and translation of several mRNAs (Bagni et al., 2012). One report suggested FMRP might also function as splicing enhancer (Didiot et al., 2008). Additionally, Drosophila FMRP has been related to the RNA-editing pathway (Bhogal et al., 2011).

### Regulation of mRNA transport

FMRP transports RNA/mRNAs from the cell body to synapses in an activity-dependent manner and through a dynamic association with microtubule motors (Kanai et al., 2004; Antar et al., 2005; Ferrari et al., 2007; Dictenberg et al., 2008; Charalambous et al., 2013). FMRP granules transport mRNA including its own (Antar et al., 2004; Ferrari et al., 2007; Kao et al., 2010), and the absence of FMRP impairs the localization of *Map1b* and *SAP90/PSD-95-associated protein 4* (*Sapap4*) mRNAs, thus altering the proper synthesis of these proteins at synapses (Dictenberg et al., 2008; Kao et al., 2010).

### Regulation of mRNA stability

Initial studies performed in *Fmr1* KO mice have revealed that the absence of FMRP alters the abundance of hundreds of mRNAs in the brain (Brown et al., 2001; Miyashiro et al., 2003; Gantois et al., 2006); a few mRNAs were found to be down regulated in all three studies. Further analyses on specific mRNAs showed that dysregulation occurred in specific brain areas and/or subcellular compartments, suggesting that FMRP might regulate the same mRNA in multiple ways (Miyashiro et al., 2003). FMRP modulates the stability of certain mRNAs by preventing or sustaining mRNA decay (De Rubeis and Bagni, 2010). As an example of the two opposite activities on different mRNAs, it has been shown that hippocampal FMRP protects *PSD-95* mRNA from decay (Zalfa et al., 2007) in an activity-dependent manner; however, FMRP protein also facilitates the decay of *nuclear RNA export factor 1 (NXF1)* mRNA in mouse neuroblastoma (N2a) cells (Zhang et al., 2007). Furthermore, FMRP regulates *PSD-95* mRNA stability in the hippocampus (Zalfa et al., 2007) and regulates translation at cortical synapses (Muddashetty et al., 2007). *PSD-95* mRNA is an important player in synaptic plasticity and is affected in ASD (Feyder et al., 2010) and SCZ (Toro and Deakin, 2005).

The cortical region of the *Fmr1* KO mouse brain shows the reduced expression of different GABA_*A*_ receptor subunits (El Idrissi et al., 2005; Gantois et al., 2006), consistent with evidence of imbalanced GABAergic signaling in FXS patients. Taken together, FMRP-RNPs might play different roles in several brain regions and regulate mRNAs through different mechanisms according to the developmental stage and subcellular localization.

### Regulation of mRNA translation

The translational dysregulation of FMRP mRNA targets significantly contributes to the FXS phenotype (Bagni et al., 2012; Darnell and Klann, 2013). Initial studies performed in lymphoblastoid cells derived from FXS individuals showed an increased translation rate in several FMRP targets (Brown et al., 2001). The increased translation of FMRP mRNA targets was also observed in *Fmr1* KO mice specifically at synapses, consistent with the idea that FMRP functions as a repressor of translation (Muddashetty et al., 2007; Narayanan et al., 2007; Napoli et al., 2008; De Rubeis et al., 2013).

FMRP activity is regulated in response to different receptor signaling cascades, i.e., type I metabotropic glutamate receptors (mGluRs) (Huber et al., 2002), the 2-amino-3-(5-methyl-3-oxo-1,2-ox-azol-4-yl) propanoic acid (AMPA) receptors (Nakamoto et al., 2007), the γ-aminobutyric acid (GABA) receptors (Centonze et al., 2008; Curia et al., 2009; Shang et al., 2009), the N-methyl-D-aspartate (NMDA) receptors (Suvrathan et al., 2010; Yun and Trommer, 2011; Eadie et al., 2012), the tyrosine kinase or BDNF/NT-3 growth factor (TrkB) receptors (Napoli et al., 2008; Louhivuori et al., 2011; De Rubeis et al., 2013), the dopamine (DA) receptors (Wang et al., 2008) and recently the cannabinoid receptors (Maccarrone et al., 2010; Busquets-Garcia et al., 2013).

One of the most affected and best characterized signaling cascades in fragile X is the mGluR (Bear et al., 2004). Upon mGluR receptor activation, FMRP-mediated translational block is released and protein synthesis can ensue. In the absence of FMRP, the increase in protein synthesis results in a receptor imbalance; an increase in the mGluR1 and mGluR5 activity and the reduced insertion of AMPA receptors at the surface that leads to enhanced mGluR long-term depression (mGluR-LTD) (Bear et al., 2004).

mGluR-LTD is a form of synaptic plasticity that involves mRNA targeting and local protein synthesis and degradation (Bear and Malenka, 1994), and this condition can be induced through the application of (S)-3,5-dihydroxyphenylglycine (DHPG) (Wisniewski and Car, 2002) in a protein synthesis-independent manner (Huber et al., 2002). In *Fmr1* KO mice, DHPG-induced LTD is strongly increased and these electrophysiological phenotypes established the “mGluR theory” in FXS (Bear et al., 2004).

FMRP activity is regulated through posttranslational modifications. DHPG-induced LTD also activates FMRP synthesis at synapses (Antar et al., 2004; Ferrari et al., 2007; Kao et al., 2010), which in turn is quickly degraded through the ubiquitin-proteasome system (Hou et al., 2006). The effect of FMRP on protein synthesis is influenced by the phosphorylation status of FMRP (Ceman et al., 2003), via the mTOR pathway (Narayanan et al., 2007): phosphorylated FMRP represses translation, while dephosphorylated FMRP releases the inhibition, allowing protein synthesis to ensue, a mechanism similarly shown for previously characterized eukaryotic initiation factor 4E binding proteins (eIF4E-BPs) in non-neuronal cells (Richter and Klann, 2009).

FMRP has also been detected in P bodies (PB), stress granules (SG) (Kedersha et al., 2005), and cytoplasmic structures, containing translationally silent pre-initiation complexes. FMRP is part of mRNPs (Siomi et al., 1996; Laggerbauer et al., 2001; Ishizuka et al., 2002; Zalfa et al., 2003; Anderson and Kedersha, 2006; Monzo et al., 2006; Papoulas et al., 2010; Charalambous et al., 2013), supporting the function of FMRP as a translational repressor at the initiation level, as observed at synapses both *in vitro* (Laggerbauer et al., 2001) and *in vivo* (Napoli et al., 2008; De Rubeis et al., 2013).

We have shown that FMRP represses translation through its binding to CYFIP1, a neuronal eIF4E-BP (Napoli et al., 2008). CYFIP1 binds to eIF4E, blocking the initiation of translation. Subsequently, the synaptic stimuli CYFIP1-FMRP complex is released from eIF4E and translation ensues (Napoli et al., 2008). Notably, CYFIP1 is also implicated in actin cytoskeleton remodeling (Kobayashi et al., 1998; Eden et al., 2002; Schenck et al., 2003; Stradal et al., 2004; Chen et al., 2010). We have recently shown that CYFIP1 links local protein synthesis and actin dynamics (De Rubeis et al., 2013). FMRP has also been proposed to regulate mRNA elongation (Darnell et al., 2011).

## FXS and commonalities with other diseases

FXS is the most common monogenic cause of ASD, and 30% of patients with FXS present autistic behaviors (Bagni et al., 2012). Early studies performed on heterozygous females carrying the fragile X mutant gene showed a greater frequency of psychopathologies associated with schizophrenia spectrum diagnoses (Reiss et al., 1988). Furthermore, carriers of premutated *FMR1* alleles (reduced FMRP levels) have been associated with a significant degree of psychiatric disorders (Bourgeois et al., 2009). Recently, low FMRP levels have been detected in the postmortem brain from subjects with SCZ, BD and MDD (Fatemi et al., 2010; Kelemen et al., 2013; Kovacs et al., 2013) and in blood samples from schizophrenia patients (Kovacs et al., 2013). Some individuals that display psychoses also carry FMR1 full and pre-mutations (Jonsson et al., 1995; Ashworth et al., 1996; Khin et al., 1998).

It is not known whether decreased levels of FMRP are the cause or the consequence of the development of these disorders. However, it is tempting to speculate that the loss or reduced function of FMRP might lead to a dysregulation of particular FMRP target genes associated with ASD, SCZ, and MD, suggesting the correlation of certain FXS features with these neuronal disorders. Because the GABAergic system is dysfunctional in these disorders (Kelemen et al., 2013) and the lack of FMRP affects the expression of some GABA receptor subunits (D'Hulst and Kooy, 2007), it is reasonable to hypothesize that FMRP reduction might explain the alterations of proteins associated with the GABAergic system in these different neurological diseases. Indeed, recent findings showed that a selective activator of GABA_*B*_ receptor reversed some FXS associated pathologies (Henderson et al., 2012).

It cannot be ruled out that certain proteins, which are risk factors for ASD, SCZ and/or MD, work together with FMRP and might disrupt the function of this protein in a disease context. Recently, it has been observed that topoisomerase Top3β, a risk gene for SCZ and ASD (Iossifov et al., 2012; Xu et al., 2012; Stoll et al., 2013), binds to FMRP and modifies the function of this protein *in vitro*, thereby supporting normal neurodevelopment and averting mental disorders (Xu et al., 2013). In addition, the authors observed that the disruption of either *Top3*β or *Fmr1* genes in Drosophila led to a dysregulation of *ptk2*, which is genetically associated with SCZ (Walsh et al., 2008). Notably, CYFIP1 has been associated with ASD (Sahoo et al., 2006) (Doornbos et al., 2009; Van Der Zwaag et al., 2010; Von Der Lippe et al., 2010; Talebizadeh et al., 2013), SCZ and epilepsy (Sahoo et al., 2006; Tam et al., 2010; Zhao et al., 2012). In addition, we have recently shown that the CYFIP1 interactome contains many novel proteins associated with ASD, SCZ, and MDD, providing new perspectives to define the regulatory pathways shared by neurological disabilities characterized by spine dysmorphogenesis (De Rubeis et al., 2013), a common feature of several neuropsychiatric disorders (Penzes et al., 2011).

Over the last 10 years, several hundred putative FMRP mRNA targets have been identified in the brain (Brown et al., 2001; Chen et al., 2003; Miyashiro et al., 2003; Zalfa et al., 2003, 2007; Muddashetty et al., 2007; Darnell et al., 2011), and more than 6000 targets have been identified in non-neuronal cells (Ascano et al., 2012). While these analyses have expanded the number of FMRP targets, further studies are required to elucidate the extent to which each mRNA contributes to the FXS clinical phenotype/s. There is substantial evidence that individuals with intellectual disabilities are prone to psychological profiles independently of the genetic and/or environmental cause (Turk, 2011).

Based on several large-scale studies, the number of FMRP neuronal target mRNAs is approximately 1,400 (Brown et al., 2001; Chen et al., 2003; Miyashiro et al., 2003; Darnell et al., 2011). We compared 1,169 unique (non-overlapping in the mentioned studies) FMRP mRNA targets with *de novo* ASD associated genes identified through recent genome-wide association studies (GWAS)(Iossifov et al., 2012; Neale et al., 2012; O'roak et al., 2012a,b; Sanders et al., 2012), obtained from the SFARI (http://gene.sfari.org) and the Autism databases (AutDB (http://www.mindspec.org/autdb.html).

**Figure 2:**
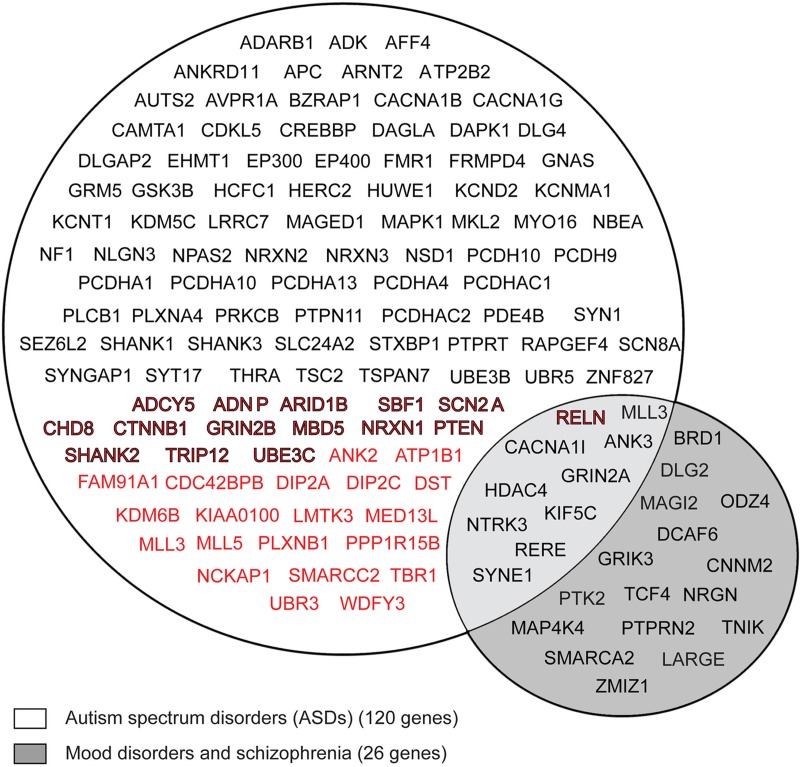
**Venn diagram of FMRP mRNA targets associated with autistic spectrum disorder (ASD), schizophrenia (SCZ) and mood disorder (MD).** FMRP neuronal target genes (1169) were compared with *de novo* gene disruptions (nonsense, splice and frameshifts) reported in GWAS are associated with ASD, as previously described (Iossifov et al., 2012; Neale et al., 2012; O'roak et al., 2012b; Sanders et al., 2012), the 528 genes from the SFARI (https://sfari.org/resources/sfari-base) and the 304 genes from the AutDB databases (http://autism.mindspec.org/autdb/Welcome.do). Approximately 120 FMRP target genes were associated with ASD (in black). Among the 120 target genes, included 35 genes were identified from the GWAS (in red) and 100 genes were identified from the two databases (in black). Fifteen genes showed overlap between the GWAS, the SFARI, and AutDB databases (in black/red color). The 1169 FMRP target genes were compared with the genes associated with SCZ and MD in the NHGRI GWAS database (http://www.genome.gov/gwastudies/) and 26 common FMRP targets were shown.

As represented in Figure 2, according to the GWAS, 35 FMRP target mRNAs are associated with ASD (in red), while the SFARI and AutDB databases have revealed that 100 FMRP target mRNAs are candidate genes for ASD (in black). Fifteen genes overlap between the results obtained in the GWAS and the SFARI and AutDB databases (in black/red color). This analysis shows that approximately 10% of the neuronal FMRP targets identified, in the above-mentioned studies, overlap with the genes associated with ASDs (120 out of 1169).

We also compared the 1,169 FMRP target mRNAs with 176 genes associated with BD, attention deficit-hyperactivity disorder (ADHD), mood disorder (MD), and SCZ (GWAS compiled by the National Human Genome Research Institute catalog http://www.genome.gov/). Twenty-six (out of 176) FMRP target mRNAs were also identified in this cohort (Figure 2, in gray). Because a few genes in this group (10) were also detected among the FMRP targets in the ASD group, it is reasonable to hypothesize that ASD, SCZ, and mood disorders (BD, MDD, ADHD) share certain common signaling pathways.

## Future perspectives

Post-transcriptional studies have revealed that the FMRP regulon controls disease-related proteins that affect both neurodevelopment and adult brain plasticity and homeostasis. The emerging wave of genetic association studies has revealed a large number of risk genes for several neurodegenerative diseases and neurodevelopmental disorders, such as SCZ, ASD, and BD (http://www.genome.gov/gwastudies).

The risk genes for neurodevelopmental disorders, identified through GWAS, were compared with the list of the FMRP targets, and the results suggest that several pathways are dysregulated in FXS and might account for specific FXS phenotypes.

As the FMRP acts as a protein synthesis repressor, it is reasonable to propose that the FXS phenotype might reflect the overexpression of specific genes. However, FMRP not only regulates gene expression at the translational level, but it also influences the stability of several mRNAs. Furthermore, to determine the functional association of the FMRP regulon with the repertoire of genes altered in individuals carrying ASD, SCZ, and MD, it is important to investigate the dosage of these genes in individuals with FXS. Moreover, FXS is a neurodevelopmental disorder, and the absence of FMRP could affect the expression of specific targets at different developmental stages and in different brain areas. Further studies on FMRP targets and the FMRP interactome at specific developmental stages would help to determine the cause of these disorders and develop further strategies to ameliorate FXS.

### Conflict of interest statement

The authors declare that the research was conducted in the absence of any commercial or financial relationships that could be construed as a potential conflict of interest.
